# Increasing incidence rate of breast cancer in cystic fibrosis - relationship between pathogenesis, oncogenesis and prediction of the treatment effect in the context of worse clinical outcome and prognosis of cystic fibrosis due to estrogens

**DOI:** 10.1186/s13023-023-02671-z

**Published:** 2023-03-20

**Authors:** Nela Stastna, Kristian Brat, Lukas Homola, Audun Os, Dagmar Brancikova

**Affiliations:** 1 Department of Respiratory Diseases, University Hospital Brno, Jihlavska 20, Brno, 62500 Czech Republic; 2 Faculty of Medicine, Masaryk University Brno, Kamenice 5, Brno, 62500 Czech Republic; 3 Department of Children’s Infectious Diseases, University Hospital Brno, Jihlavska 20, Brno, 62500 Czech Republic; 4 Department of Pulmonary Medicine, Oslo University Hospital, Kirkeveien 166, Ullevål, Oslo, 0450 Norway; 5 Department of Hematology, Oncology and Internal Medicine, University Hospital Brno, Jihlavska 20, Brno, 62500 Czech Republic

**Keywords:** Cystic fibrosis, Breast cancer, Estrogens, Anticancer treatment, CFTR modulator therapy

## Abstract

Cystic fibrosis (CF) is the most common genetic disease in the Caucasion population. Thanks to the CFTR modulators therapy, life expectancy will significantly improve. New therapeutic challenges can be expected, including diseases associated with ageing and higher incidence of cancer, as evidenced by recent epidemiological studies. The increasing incidence of tumors includes also breast cancer. The risk of breast cancer is higher in CF patients compared to the general population. Sex hormones, especially estrogens, also affect on the pathophysiology and immunology of the CF. Previous research, has demonstrated unequivocal survival rates for female CF patients compared to their male counterparts. Is demonstrated, that chemotherapy used for breast cancer affects the CFTR channel and CFTR modulator therapy has frequent side effects on breast tissue. In this review, we focus on the effects of female sex hormones on CF disease, pathophysiological relationships between CF and breast cancer, and the impact of antitumor treatment on both, malignant disease and CF. The potential for further investigation is also discussed.

**Supplementary Information:**

The online version contains supplementary material available at 10.1186/s13023-023-02671-z.

## Background

Cystic fibrosis (CF) is the most common rare multiorgan disease caused by a mutation in the CFTR (cystic fibrosis transmembrane conductance regulator) gene, which regulates the CFTR protein, a cyclic adenosine monophosphate-regulated Cl^−^ channel. The most common mutation affecting the function of the CFTR protein is *F508del*, a target for modern pharmacotherapy. The European Cystic Fibrosis Society 2019 annual report (a year before the widespread introduction of CFTR modulators in clinical practice) stated that the median survival was 19 years, as only about 25% of all patients were older than 30.9 years [1]. Patients treated with CFTR modulators have a longer life expectancy. In Canada, the median survival was estimated to be 52.3 years of age by 2017 [2]. With improvements in the probability of longer survival, an increased incidence of breast cancer is expected in female CF patients. Breast cancer was not reported as a common type of cancer in CF patients, however, increasing trends in incidence were observed in recent epidemiological studies. Currently, breast cancer is the most common cancer disease after gastrointestinal tumors (colon, rectal, bowel, ileum) [3]. In a general population, the life expectancy of males is shorter than of females, however, in the CF population, this epidemiological parameter is reversed. The Canadian CF Registry Annual Data Report 2017 indicated the median survival age of 49 years for females and 56 years for males [2]. The gender gap occurs only after the age of 15, until then the mortality rates of both sexes are comparable [4]. This supports the hypothesis that reproductive hormones may affect the prognosis of the disease. Despite women with CF have comparable estradiol serum concentrations to non-CF women, several studies reported that estrogens play a different role in CF than in the general population [5]. Sex hormones affect the expression of the CFTR channel, modulate ion transport, affect the immune system and significantly worsen the course of the disease and its prognosis [6].

## Methods of literature review

For this review, we performed an extensive literature search using PubMed, Web of Science and Scopus databases between May and July 2022 for peer-reviewed, English-language, original research studies, human and animal, pediatric and adults with CF, in vivo and in vitro, observational studies, case studies, treatment or diagnosis guidelines, reviews, and systematic reviews. Studies published between January 1st, 1990 and July 29th, 2022, were included. We searched for the following keywords: cystic fibrosis, CFTR, mucoviscidosis, breast cancer, breast carcinoma, breast tumor, breast neoplasia, breast malignancy, mammary carcinoma, mammary cancer, mouse, animal, rat, human, chemotherapy, anticancer treatment, cytostatic treatment, estrogen, hormonal treatment, CFTR modulator, ivacaftor, tezacaftor, lumacaftor, elexacaftor. Meta-analyses and non-peer-reviewed publications were excluded. Duplicate citations and articles of inappropriate types, publication dates, or language were removed from the primary selection. A secondary selection of titles and abstracts eliminated inappropriate study population (non-CF, non-*F508del* carriers). Final selection of titles and abstracts was restricted to articles relevant to the fields of breast cancer, female health and cystic fibrosis. Outcomes for the coincidence of cystic fibrosis and breast cancer were evaluated from the included studies. The purpose of this review was to provide a comprehensive overview of studies and study outcomes from the field of breast cancer and cystic fibrosis in a non-statistical manner.

## Epidemiology of breast cancer in patients with cystic fibrosis

### Epidemiological studies of patients with cystic fibrosis

Garcia et al. evaluated the mammary histomorphology of 19 postpubertal women with CF [7]. They reported two cases of an invasive mammary carcinoma, ductal type, low grade and one case of an invasive ductal type of high grade. Previous epidemiologic studies have demonstrated a low incidence of breast cancer. A study of cancer risk among patients with CF in North American countries was conducted between 1985 and 1992 and compared with European registeries. The authors reported 41 cases of tumor, with only 2 cases of breast carcinoma, while in Europe there was no case of breast cancer in the overall 41 tumor cases [8]. The number of reported cases was compared to the number of expected cases (calculated from population-based data on cancer incidence) and breast cancer had a low observed-to-expected ratio (0.8) [8]. Most of the cases manifested over 30 years of age. In an other study with more than 40,000 CF patients, 172 cases of cancer were reported between 1990 and 2009 [9]. The authors reported a declining incidence trend in the second decade of the study period compared to the first decade (standardized incidence ratio 0.7, CI 95%) [9]. In contrary, Archangelidi et al. reported opposite trends in cancer epidemiology. The authors compared data from the CF registry in UK with data from the general population between 1999 and 2017 [3]. As a result, 146 cases of malignancy were identified, of these, 13 cases of breast cancer. This means a rate of 46.5/100,000 cases (95%, CI: 33-59.8) in the CF population vs. 14.5/100,000 in the general population. The results of this study should be interpreted with precaution due to the small number of patients. No increased risk was seen in association with homozygosity for *F508del* mutation. The fact that heterozygosity for CF gene is not a risk factor for breast cancer was also confirmed in a Swedish study where CF patients and their closest relatives were followed [10]. However, this study didn’t record any case of breast cancer. In the Austrian CF center between 1995 and 2019, they recorded 11 cases of malignant disease in 229 patients [11]. Although they did not record any cases of breast cancer, it is interesting that of these 11 cancer patients, 10 were women. Despite the number of patients with breast cancer was small, a trend towards increasing incidence could be seen over time (Table 1).

**Table 1:** An overview of epidemiological studies among CF patients

Study	Country of origin	Study period	No. CF patients	No. cases of all cancer types	No. breast cancer
Neglia JP	USA, CAN, EU*	1982–1994	> 46,511	82	2
Garcia FU	USA	1971–1997	19	unobserved	3
Johannesson M	SWE	1968–2003	884	41	0
Maisonneuve P	USA	1990–2009	41,188	172	15
Archangelidi O	GBR	2009–2017	12,886	146	13
Appelt D	AUT	1995–2019	229	11	0

**Legend**: USA: The United States of America, CAN: Canada, EU: European Union, SWE: Sweden, GBR: The United Kingdom of Great Britain and Northern Ireland, AUT: Austria

* Austria, Belgium, Denmark, France, Finland, Germany, Hungary, Iceland, Ireland, Italy, The Netherlands, Norway, Romania, Spain, Sweden, Switzerland, UK

### Epidemiological studies on risk of breast cancer in patients harboring the *F508del* mutation

A recent study by Shi et al., using the data of the UK biobank (almost 80,000 subjects with any cancer diagnosis) demonstrated that the prevalence of *F508del* carriers was significantly higher in patients with cancer (3.29% vs. 3.15%) compared to patients without cancer. The 3.18% carrier rate for breast cancer was not statistically significant (P = 0.37) [12]. The rate of carriers was similar for both genders. The subjects included in this study were only carriers of the *F508del* mutation and the median age was rather higher compared to the median life expectancy of patients with CF. The risk of breast cancer before the age of 40 didn´t differ in *F508del* CFTR mutation carriers and non-carriers (Table 2) [13].

**Table 2:** An overview of epidemiological studies among *F508del* carriers

Study	Country of origin	Study period	No. F508del carriers	No. breast cancer
Shi Z	GBR	2006–2010	15,118	499
Southey MC	AUS	1992–1995	272	6

**Legend**: GBR: The United Kingdom of Great Britain and Northern Ireland, AUS: Australia

## The severity of breast cancer

### Distribution of breast cancer in the general population

By 2020, breast cancer was the most prevalent cancer disease and the leading cause of cancer-related deaths among women worldwide [14]. Breast cancer is rare at a young age, while the incidence rates increase with advancing age in postmenopausal women [14]. Regions with higher GDP are most affected [14]. The numbers of male breast cancers are minimal.

### Physiology of estrogens and their relationship to the development of breast cancer

An important risk factor for breast cancer development is exposure to estrogens. They occur in three biochemical forms: 17β-estradiol (E2) in the largest quantity in pre-menopausal women with the highest affinity for its receptors (ERs), estrone and estriol. The biological action of estrogens is mediated by binding to ERs. The tissues most exposed to estrogens such as the breast, cervix and prostate, are also the most common organs affected by cancer disease [14]. Estrogens have multiple functions in mammary cells, as they regulate the growth and differentiation of the cells and they influence on physiology and pathophysiology of the reproductive process. They potentiate cell proliferation, thus influencing the rate of spontaneous mutations. Tumor cells produce various types of interleukins and prostaglandins, which significantly increase estrogen production [15, 16]. Epidemiological studies have established an association between breast cancer and hormonal risk factors (nulliparity, higher age at first birth, less breastfeeding etc.) [14]. Decreased estrogen levels or efficacy are thought to reduce the risk of cancer and prolong patient’s life. Hormone therapy for breast cancer uses this principle. About 75% of all breast cancers are ER+ [15].

## Effect of estrogens on the pathophysiology of CF and breast cancer

### Relationship between endogenous and exogenous changes in estrogen levels and breast cancer

The risk of breast cancer is hormone dose-dependent and increases with higher level of endogenous sex hormone serum level, exogenous hormone such as oral contraceptives (OCPs) and post-menopausal hormone replacement therapy (HRT) [17]. Women with CF underutilize OCPs compared to the general population [18]. Women with late onset of menarche and early menopause, lower age at first birth, higher number of children and prolonged breastfeeding, no excess body weight have lower chance of developing breast cancer due to shorter exposure to sex hormones [14]. Menarche in CF girls starts at the same age or slighly later that in healthy girls [18]. The menopause in CF and usage of HRT has not been studied properly yet, the onset of menopause hasn’t been studied. Women with CF got pregnant less frequently compared to non-CF women [18]. Thus, the number of ovulatory cycles producing estrogens is higher.

### Mechanisms of estrogen action

The gender gap could be partly explained by modulation of inflammatory responses by female sex hormones. Breast tumors proved to be autonomous in retaining a constant estradiol concentration.

The expression of estrogen syntetase (aromatase) mRNA is enhanced in breast cancer [16]. The levels of E2 in breast tumor tissue are significantly higher compared to their serum concentration [16]. Aromatase increases estradiol synthesis which binds to ER that, in turn, promotes luminal cancer cell proliferation. The breast tumor epithelial cells induce prostaglandin E_2_, tumor necrosis factor α (TNFα) and other cytokines production, which regulate the expression of aromatase [16]. In breast cancers, the expression of TNFα correlates with higher tumor cell proliferation, higher grade of malignancy, increased tendency to metastasis and a general poor prognosis [16]. The expression of TNFα is induced by nuclear factor (NF)-κB [16]. NF-κB controls cell survival. Defect in NF-κB results in increased susceptibility to apoptosis. Blockade of NF-κB may result in loss of tumor cells proliferation, apoptosis or may enhance sensitivity to anticancer drugs. ERs can supress NF-κB by different mechanisms, e.g. E2 mediates upregulation of secretory leucoprotease inhibitor which inhibits NF-κB [16, 19]. CFTR expression inversely correlates with NF-κB activity; defect or suppression of the CFTR results in abberrant activation of NF-κB [20]. The carcinogenic effect of estrogens is multidirectional. Estrogens promote proliferation via ER-mediated pathway (e.g. NF-κB interaction with ERs), ER-independent phosphorylation of target molecules and via direct genotoxic effects of estrogen metabolites on DNA damage, mutation and cell transformation [17].

### Relationship between estrogens and CFTR expression

Estrogens affect CFTR expression. The expression of CFTR mRNA and protein is changing during the ovulatory cycle. In the folicular phase, the expression of CFTR is enhanced [21]. An in vivo study demonstrated CFTR can be up-regulated under estrogen stimulation [22]. Increased estrogen level during the ovarian hyperstimulation syndrome leads to upregulation of CFTR and enhanced CFTR channel activity [23]. CFTR expression is not related to ER status [20].

## Breast cancer in patients with cystic fibrosis

### The importance of estrogen receptors

There are two subtypes of ERs – ER α and ERβ. ERs are activated by E2. ERα regulates the genes involved in cell proliferation, differentiation and migration and deregulated actions of ERα are associated with breast cancer [15]. ERβ plays an important role in epithelial differentiation and acts as a tumor suppressor [17, 19]. Inhibition of the ERα has become one of the main strategies for the prevention and treatment of breast cancer. Chotirmall et al. demonstrated that patients with CF have significantly higher expression of ERα and ERβ when compared to non-CF subjects and that ERβ expression is significantly higher in CF than of ERα [19].

### Relation of CFTR expression to breast cancer development

Abnormal expression of CFTR has been observed in several types of cancer, however it is unknown if it affects the function of tumor-supressor or tumor-promoting genes. CFTR regulates cell proliferation and invasion, suggesting CFTR might be related to the oncogenesis of malignant tumors [20]. Suppression of CFTR function by its inhibitors promotes cell invasion and migration [20]. There is no significant difference in CFTR expression levels between invasive ductal carcinoma and invasive lobular carcinoma [20]. CFTR expression is believed to be regulated by TNFα [24]. The mRNA level of CFTR is downregulated and the CFTR protein level is decreased in breast cancer samples that associates with poor prognosis [20]. Therefore, the CFTR expression level could be used as a new prognostic biomarker.

### Relation of CFTR and breast cancer metastasis

Suppression of the CFTR function significantly inhibits the expression of E-catherin gene and leads to the loss of E-catherin protein [20]. This promotes epithelial-mesenchymal transition which is an important step in the process of metastasis. Epithelial cells lose cell polarity, cell-cell adhesions and gain migration and invasive properties to become mesenchymal stem cells. The ability to invade and metastasize is inversely correlated with E-catherin level. Knockdown of the CFTR gene enhances the expression of urokinase-type plasminogen activator, a serine protease that plays a central role in extracellular matrix degradation, invasion, regulation of epithelial-mesenchymal transition and metastasis during cancer [20]. Urokinase-type plasminogen activator is known to be activated by NF-κB and defective CFTR has been shown to result in endogenous activation of NF-κB [20]. In contrary, overexpression of the CFTR suppresses the epithelial-mesenchymal transition and invasive properties of cells [20].

### Possible anticarcinogenic effect of the CFTR protein

On the other hand, several studies suggested an opposite effect of CFTR on oncogenesis. CFTR mutation may be cancer protective as elevated extracellular ATP (adenosine triphosphate) is known to inhibit breast cancer cell line growth in vitro and in vivo [12, 25]. As CFTR belongs to the ATP-binding cassette transporter family, CFTR mutation would result in higher concentrations of serum ATP. CFTR knockout mice model showed reduced breast cancer implantability and decreased growth rate [12, 25]. This imposes that breast cancer in CF women might be less aggressive. In the real life, however, the reviewed tumors were grade III and poorly diferentiated in a study of 272 cases [12].

## Relation of CFTR modulators, breast health and breast cancer outcomes (including anticancer therapy)

### Effect of CFTR modulators on the mammary gland

Side effects on the breast tissue have been observed during CFTR modulator use, mostly in adult patients. Breast disorders like breast mass, breast inflammation, gynecomastia and nipple disorders have been described in both sexes [26]. A case report demonstrated breast development in a 7 year-old CF girl treated with ivacaftor as a rare dose-dependent side effect [27]. The levels of E2, follicle-stimulating hormone and luteinizing hormone were appropriate for age, while the Tanner score was III-IV. Ultrasound of the breasts showed symmetrical development of glandular breast tissue with no structural abnormalities. After the ivafactor treatment discontinuation, a fast regression of the breasts has been observed, back to Tanner I. This side effect was dose dependent, as no recurrence of breast development was observed with a lower-dose ivacaftor treatment restart. [27].

Elexacaftor, tezacaftor and ivacaftor are also substrates to the breast cancer resistance protein (BCRP) efflux transporters, that mediate multidrug resistance [26]. The BRCPs expel many potent antineoplastic drugs and this mechanism may result in cancer treatment failure. BCRP expression was detected in breast cancer [28].

### Relation of breast cancer hormone therapy and clinical course of CF

Little is known about the effect of hormonal antitumor treatment used for mammary carcinoma cure in CF. In vitro and in vivo studies investigated the effect of E2 and tamoxifen on calcium-activated chloride channel (CaCC) Cl^−^ currents in human bronchial epithelial cells carrying *F508del* mutation. The authors reported that tamoxifen could restore CaCC currents inhibited by E2 [29]. Tamoxifen also activated the transmembrane Cl^−^ currents potentiated by ATP (a mutation independent effect) and the effect was synergistic with lumacaftor/ivacaftor [29]. In an animal study, raloxifene analog LY117018 induced CFTR expression, didn’t supress epithelial sodium channel expression and prevented fluid accumulation [30].

### Relation of CF outcomes and cytostatic treatment of breast cancer

The data on chemotherapy used in breast cancer (anthracyclines, taxanes, considered carboplatin, methotrexate) is still insufficient in the context of cystic fibrosis patients. Maitra and Hamilton evaluated the effect of doxorubicin on CFTR expression and CFTR-associated chloride secretion in epithelial cells [31]. The authors demonstrated that noncytotoxic doses of doxorubicin significantly increased CFTR expresion and chloride secretion in epithelial cells [31]. Treatment with doxorubicin significantly improved the half-life of *F508del*-CFTR due to increased resistance of *F508del*-CFTR to protease digestion [31]. Similar observation was described in a case report of a fibrosarcoma in a CF patient treated with cyclophosphamide and anthracycline, epirubicine [32]. In this case, the patient improved also lung function and a chronic *Pseudomonas aeruginosa* infection was inhibited [32]. Another case report demonstrated successful treatment of osteosarcoma treated by cisplatin, doxorubicin and methotrexate [33]. Similarly to previous reports, the patient‘s pulmonary functions improved during the chemotherapy despite the fact that the treatment course was complicated by pulmonary exacerbations caused by *Pseudomonas aeruginosa*, *Staphyloccocus aureus*, *Mycobacterium avium* and *Mycobacterium abscessus*. Despite the exacerbations, the eradication of nontuberculous mycobacterias was successful. This might be partly explained by the effect of doxorubicin on CFTR and the anti-inflammatory nature of methotrexate. Interestingly, methotrexate was shown to increase FEV_1_ by a median of 9% in five patients with CF treated with low dose regime [34].

## Discussion

The increasing incidence of breast cancer in CF patients is disturbing. Breast cancers in CF are most prevalent in the age category 36–45 years [3]. In the general population, the peak prevalence is in higher age [14]. Life expectancy of women with CF is expected to rise in the near future, largely due to the new era of CFTR modulators. It can be expected that the rates of oncological diseases in CF patient population will also increase. CFTR modulator therapy improves conception ability, women are pushed to use OCPs more, which is a risk factor for breast cancer. Estrogen is also produced by adipose tissue. Thus anabolic effect of CFTR modulators might influence cancer risk. Mortality related to breast cancer in CF women was not assessed sufficiently, although in the general non-CF population of women, it is a leading cause of cancer mortality [14]. Estrogen negatively affects the course of CF and mediates carcinogenesis by several mechanisms [6]. The role of NF-κB is essential, however the process of NF-κB activation and TNFα induction by estrogens and CFTR expression is contradictory [16, 19, 20]. TNFα plays a central role in initiation, promotion and metastasis of breast cancer. Suppression of the CFTR function enhances metastatic ability by several mechanisms, [20]. There is increasing interest in the association of cancer incidence with the genetic variation of CFTR. Although several studies demontrated that *F508del* mutation is considered a risk factor for developing cancer, the role of CFTR in relation to oncogenesis is unclear [11–13, 20, 25]. CFTR is downregulated in breast cancer tissue [20]. CFTR downregulation correlates with poorer clinical prognosis of patients with mammary carcinoma [20]. CFTR might be useful in considerations of which patients might benefit from adjuvant therapy.

We still lack data and knowledge whether hormonal therapy, which is a frequent therapeutic choice, has the same effectiveness in CF as in the non-CF population. In contrast, chemotherapy, specifically anthracyclines have shown to increase the expression of CFTR and improve its stability [31]. This might be beneficial to CF patients as also reported in a few case reports [32]. The cytotoxic nature of anthracyclines and adverse effects associated with their chronic use make this class of compounds unsuitable for clinical use, because they were designed to maximize cytotoxicity for cancer chemotherapy. Development of anthracycline analogs that promote functional expression of *F508del*-CFTR without toxicity may provide a clinical benefit in CF. Methotrexate, has the advantage of its anti-inflammatory effect. Hormonal targeted therapies in breast cancer like selective estrogen receptor modulators (i.e. raloxifene, tamoxifen) increases CFTR expression [30]. They can also reduce the risk of osteoporosis by increasing bone mineral density in post-menopausal women. Tamoxifen might be a curative, mutation-independent treatment of CF due to enhancement of the Cl^−^ channel [29]. ERα and ERβ are expressed significantly higher in CF compared to non-CF subjects and ERβ expression is significantly higher in CF than that of ERα [19]. Therefore, the potential effect of the treatment is difficult to predict and should be further studied. As CFTR modulators affect the mammary gland and the effect of the rest of the drugs is unclear, further research is needed to understand the links between the therapy and the increasing incidence of breast cancer [26, 27].

## Conclusion

This article points to the links between the pathophysiology of CF, cancer and the treatment options. The incidence of breast cancer is worrying and as survival improves, cancer is likely to become a more prevalent complication of CF.

## Electronic supplementary material

Below is the link to the electronic supplementary material.

Supplementary Material 1

Supplementary Material 2

## Data Availability

Not applicable.

## List of Abbreviations

CF: Cystic fibrosis
CFTR: Cystic fibrosis transmembrane conductance regulator
E2: 17β estradiol
ER: Estradiol receptor
OCP: Oral contracentive
HRT: Hormonal replacement therapy
TNFα: Tumor necrosis factor α
NF-κB: Nuclear factor–κB
BCRP: Breast cancer resistence protein
CaCC: Calcium-activated chloride channel
