# Supplementary material for: Increasing incidence rate of breast cancer in cystic fibrosis - relationship between pathogenesis, oncogenesis and prediction of the treatment effect in the context of worse clinical outcome and prognosis of cystic fibrosis due to estrogens
Source: Orphanet J Rare Dis. 2023 Mar 20;18:62. doi: 10.1186/s13023-023-02671-z (PMC10029289; doi:10.1186/s13023-023-02671-z)
Supplement: Supplementary file 1 — Supplementary Material 1 [file 13023_2023_2671_MOESM1_ESM.docx]

Nela Stastna

Jilova 142/30A

Brno 63900

Czech Republic

November 3rd, 2022

The Editor

Orphanet Journal of rare Diseases

Subject: Increasing Incidence Rate of Breast Cancer In Cystic Fibrosis - Relationship Between Pathogenesis, Oncogenesis and Prediction of the Treatment Effect in the Context of Worse Clinical Outcome and Prognosis of Cystic Fibrosis due to Estrogens

Respected Sir,

With great admiration and belief in the articles of your journal, I seek to bring to your kind notice the rapidly increasing risk of oncological diseases in patients with cystic fibrosis, especially breast cancer.

Recently there were published several epidemiological oncological studies on cystic fibrosis (e.g. Archangelidi O., Incidence and risk factors of cancer in individuals with cystic fibrosis in the UK; a case-control study. *J Cyst Fibros* 2021). The incidence of breast cancer is reported to be rising and in a near future, thanks the CFTR modulator therapy, the survival rate will increase, the breast cancer start to be a severe cause for concern. Even now the studies showed a higher incidence in a CF cohort, than in a general population. Because it is a leading mortality cause in non-CF people, this review focus on the possible explanation of the relationship between oncological and CF disease. Also the view of CFTR modulator therapy's effect on breast tissue is discussed as a possible cause of malignant disease and the effect of chemotherapy of CF.

**Declaration of ethics approval and consent to participate:** Not applicable.

**Declaration of competing interests:** The autors declare that they have no competing interests.

**Declaration of funding:** Publication will be supported by the Czech Pneumological and Phthisiological Society (open access publication fee grant).

**Declaration of authorship contribution:** All authors read and approved the final manuscript.

This work hasn’t been published or submitted for publication nowhere else.

Yours Sincerely,

Nela Stastna

[Stastna.nela@fnbrno.cz](mailto:Stastna.nela@fnbrno.cz)

+420 732 115 426

University Hospital Brno, Respiratory Department

Jihlavska 20, Brno 62500 Czech Republic

Masaryk University, Faculty of Medicine

Kamenice 5, Brno 62500, Czech Republic
