# Supplementary material for: Increasing incidence rate of breast cancer in cystic fibrosis - relationship between pathogenesis, oncogenesis and prediction of the treatment effect in the context of worse clinical outcome and prognosis of cystic fibrosis due to estrogens
Source: Orphanet J Rare Dis. 2023 Mar 20;18:62. doi: 10.1186/s13023-023-02671-z (PMC10029289; doi:10.1186/s13023-023-02671-z)
Supplement: Supplementary file 2 — Supplementary Material 2 [file 13023_2023_2671_MOESM2_ESM.docx]

Response to Reviewers:

Dear editor, dear reviewers,

First of all, we would like to thank for your valuable comments and constructive criticisms regarding our manuscript. We really appreciate your time and we really believe your advice resulted in improvements to the quality of this paper.

Below, you will find all your comments and suggestions addressed by the authors point by point. Each comment is paired with a specific authors´response. In the manuscript, all changes to the text or tables are highlighted in red. We believe we have addressed all the comments in an encouraging way and that the manuscript will now be acceptable for publication in *Orphanet Journal of Rare Diseases*.

On behalf of the authors team,

thank you for your work once again,

Nela Stastna, M.D.

Department of Respiratory Diseases

University Hospital Brno and Masaryk University

Brno, Czech Republic, EU

February 15^th^, 2023

**Reviewer #1:**

*Cystic fibrosis (CF) is the most common genetic disease in the Caucasion population. The risk of breast cancer is higher in CF patients compared to the general population. But, A review of epidemiological studies in TABLE 1 didn't give us this conclusion. No. of breast cancer is very rare, except in the study of Shi, why? please give the explanation.*

**Authors´ response:**

We would like to thank the reviewer for this important comment. We believe this was only a misunderstanding.

Higher incidence of breast cancer in CF cohorts (when compared to the general population) has recently been observed by *Archangelidi* et al. in a case-control study. The authors reported 13 patients with breast cancer, indicating a rate of 46.5 cases per 100,000 in the CF population (95% CI, 33-59.8) compared to 14.5/100 000 in the general population. The results of this study should be interpreted with precaution due to the small number of patients.

The study by *Shi* et al. reported breast cancer incidence among *F508del* carriers (= heterozygotes, **not CF patients**) from a UK biobank, that included genetic and phenotypic data of approximately 500,000 individuals with 40-69 years of age. The authors of this study observed no difference in breast cancer incidence in heterozygote *F508del* carriers when compared to the general population (p=0.37).

To prevent confusion and for better understanding of the data, we split the original table into Table 1 (= breast cancer epidemiology in CF patients) and Table 2 (breat cancer epidemiology in heterozygote *F508del* carriers). As we can see in Table 1, the incidence of breast cancer in CF patients tends to increase over time, probably due to improved surveillance, diagnostic procedures, improved survival and improved breast cancer screening programs.

We hope the reviewer will agree.

**Reviewer #2:**

*This is an interesting review and provide us a thoughtful, content-rich information about the effects of female sex hormones on CF disease, pathophysiological relationships between CF and breast cancer, and the impact of antitumor treatment.*
*But for a review, I would like to suggest the authors use PRISMA (Preferred Reporting Items for Systematic Reviews and Meta-analyses) guidelines, or at least provide data extraction process in the article.*

**Authors´ response:**

We would like to thank the reviewer for this important comment. We completely agree that the PRISMA method would strengthen the credibility of this paper as transparent and complete reporting are essential components of high-quality research. However, we assumed that preparing the PRISMA diagram retrospectively might generate inaccuracies. In order to avoid confusion, we provided a detailed description of the literature search and the data extraction procedure in the "Methods of literature review" section. We hope the reviewer will agree.
